# Development of Ga-67 Maltolate Complex as an Imaging Agent

**Published:** 2012

**Authors:** Yousef Fazaeli, Amir Reza Jalilian, Mostafa Mohammadpour Amini, Abbas Majdabadi, Ali Rahiminejad, Fatemeh Bolourinovin, Mehraban Pouladi

**Affiliations:** a *Nuclear Medicine Research Group, Agricultural, Medical and Industrial Research School (AMIRS), Nuclear Science and Technologies Research Institute (NSTRI), Karaj, Iran.*; b *Radiopharmaceutical Research and Development Lab, Nuclear Science and Technologies Research Institute (NSTRI), Tehran, Iran.*; c *Department of Chemistry, Shahid Beheshti University, Tehran, Iran.*

**Keywords:** Ga-67, Maltolate, Imaging, Biodistribution

## Abstract

Due to the antitumor activity of Gallium MAL complex, as well as recent findings on new targeted biomolecules in malignant cells through this complex, the development of radiolabeled gallium complex for future imaging studies was targeted. Ga-67 labeled 3-hydroxy-2-methyl-4H-pyran-4-onate (Ga-67 MAL) was prepared using freshly prepared Ga-67 chloride and 3-hydroxy-2-methyl-4H-pyran-4-onate in a sodium salt form in 25 min at 40° C. The stability of the complex was checked in final formulation and human serum for 24 h followed by the administration in Swiss mice for biodistribution studies. The complex was prepared in high radiochemical purity (> 97% ITLC, > 98% HPLC) and specific activity of 13-14 GBq/mmol and was stable in the presence of serum for 48 h. The partition coefficient was calculated for the compound (log *p *= 0.40). A detailed comparative pharmacokinetic study was performed for Ga-67 cation and Ga-67-MAL. The complex is more rapidly washed out from the circulation through kidneys and liver compared to Ga-67 cation and can be an interesting tumor imaging agent due to the fact that the cold compound is undergoing clinical trials as a safe and potential therapeutic agent for cancer.

## Introduction

The interesting physical properties and the availability of gallium-67 make it an interesting nuclide for radiopharmaceutical research (1). The increasing trend in the production and use of PET radionuclides in nuclear medicine has offered new opportunities for researchers to focus on the production of new Ga-radiopharmaceuticals for feasibility studies using Ga-67 for their future PET gallium homologs. Table 1 demonstrates the most important Ga radionuclide physical properties.

Maltol (3-hydroxy-2-methyl-4-pyrone) is a naturally occurring, non-toxic compound and common food additive. Many biologically important metals form the stable complexes with maltol. Its stability arises from the easiness of deprotonation and behaving as an anionic, bidentate metal chelator.

**Table 1:** Nuclear properties of Ga radionuclides

**Properties**	^67^ **Ga**	^68^ **Ga**	^66^ **Ga**
Gamma energy (keV)	3 185 300	511(*β*+)	511(*β*+) 834 1039 2752
*β-/β*+ energy	8492	1900(*β*+)	4153(*β*+)
Mode of decay	EC to ^67^Zn	10% EC to ^68^Zn 90% *β*+	43% EC to ^66^Zn 57% *β*^+^
Nuclear reaction	^68^Zn(p,2n)^Ga-67^	^68^Ge Daughter ^66^Zn(α,2n)^68^Ge	^66^Zn(p,n)^66^Ga
Half-life	78 h	68 min	9.6 h
Natural abundance )%(	(18%)	(28%)	(28%)
possible contaminations	^66^Ga, ^65^Zn	^68^Ge	^65^Zn
Proton energy (MeV)	12-22	12-22	6-15

Tris (maltolato) gallium (III) complex has been used in the treatment against several lymphoma cell lines, including those resistant to gallium nitrate. In addition, interesting recent data demonstrated that Ga-MAL has induced the apoptosis in cells resistant to gallium nitrate, unlike gallium nitrate. Interestingly, the cellular gallium uptaken for Ga-MAL was greater with gallium maltolate than with gallium nitrate. Moreover, gallium maltolate demonstrated the inhibition on cell proliferation and induces apoptosis more efficiently than gallium nitrate (1). Other studies on gallium MAL complex have revealed potential therapeutic effects in liver and gastrointestinal cancers (2).

On the other hand, it has been recently shown that the proteasome may be a target for gallium maltolate and suggest that a radiolabeled complex can be used in imaging as reported recently(4).

Accordingly, Gallium MAL is stable in aqueous solutions at the human serum pH range (5-8) and it has significant solubility in both water and lipids proposing a possible injectable radiolabeled complex for imaging purposes.

**Figure 1:**
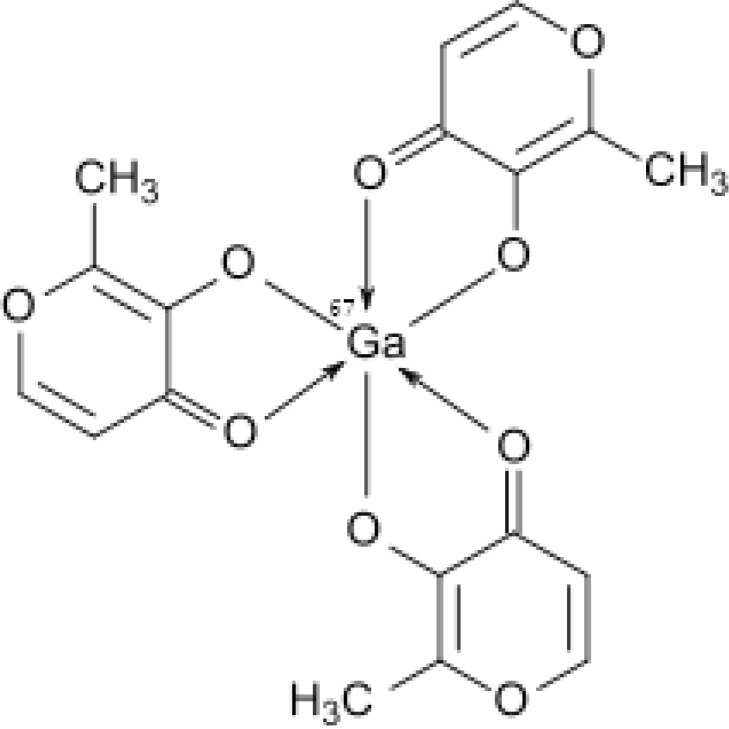
Structure of Ga-67 (III) Tris (maltolato).

However, no radio gallium labeled MAL complexes have been reported in the literature according to the author’s knowledge. Due to the interesting pharmacological properties of maltolato complexes such as solubility in serum, rapid wash-out, tumor avidity and feasible complexation with various metals (4), the idea of developing a possible tumor imaging agent using SPECT (photon emission computed tomography) through incorporating Ga-67 into a suitable anionic ligand, *i.e*. Tris (maltolato) Ga-67 (III) was investigated (Figure 1).

 Interestingly, the production and evaluation of Tris (maltolato) Ga-67 (III) for diagnostic purposes can lead to the developing ultimate Ga-68 homolog compound for PET applications.

In this work, we reported radiolabeling, partition coefficient determination, quality control and biodistribution studies (using SPECT and scarification) of Ga-67-MAL complex in Swiss mice. The time/activity diagrams for the labeled compound in vital organs have been plotted compared to the gallium cation.

## Experimental

Enriched zinc-68 chloride with a purity of more than 95% was obtained from Ion Beam Separation Group at Agricultural, Medical and Industrial Research School (AMIRS). The production of Ga-67 was performed at the Nuclear Medicine Research Group (AMIRS) 30 MeV cyclotron (Cyclone-30, IBA). Other chemicals were purchased from the Aldrich Chemical Co. (Aldrich, Germany) and the ion-exchange resins from Bio-Rad Laboratories (Canada). Thin layer chromatography (TLC) for cold compounds was performed on polymer-backed silica gel (F 1500/LS 254, 20×20 cm, TLC Ready Foil, Schleicher and Schuell, Germany). Normal saline and sodium acetate used for labeling were of high purity and had been filtered through 0.22m Cativex filters. Instant thin layer chromatography (ITLC) was performed via counting Whatman No. 2 papers using a thin layer chromatography scanner, Bioscan AR2000, Bioscan Europe Ltd. (France). The analytical high performance liquid chromatography (HPLC) used to determine the specific activity, was performed through a Shimadzu LC-10AT, armed with two detector systems, flow scintillation analyzer (Packard-150 TR) and UV-visible (Shimadzu) using Whatman PartiSphere C-18 column 250×4.6 mm, Whatman, NJ (USA). The analytical HPLC was also used to determine the specific radioactivity of the labeled compound. A standard curve was generated to calculate the mass of the final solution. Biodistribution data were acquired via counting normal saline-washed tissues after weighting on a Canberra™ high purity germanium (HPGe) detector (model GC1020-7500SL). Radionuclidic purity was checked with the same detector. For the activity measurement of the samples, a CRC Capintech Radiometer (NJ, USA) was used. All calculations and tissue countings were based on the 184 keV peak. Animal studies were performed in accordance with the United Kingdom Biological Council’s Guidelines on the Use of Living Animals in Scientific Investigations, 2^nd^ edition.

*Production and quality control of Ga-67*

Zn-68 (p, 2n) Ga-67 was used as the best nuclear reaction for the production of Ga-67. Other impurities could be removed in the radiochemical separation process according to the latest optimized reported method (6). Gamma spectroscopy of the final sample was carried out counting in an HPGe detector coupled to a Canberra™ multi-channel analyzer for 1000 sec. The chemical purity was checked through differential-pulsed anodic stripping polarography. The detection limit of our system was 0.1 ppm for both zinc and copper ions (7, 8).

*Preparation of Ga-67-MAL*

The complex was prepared in accordance with other radiogallium small complex preparation methods (9). The acidic solution (2 mL) of Ga-67 chloride (111 MBq) was transferred to a 3 mL-borosilicate vial and heated to dryness using a flow of N_2_ gas at 50-60° C. Fifty μL of sodium maltolato salt in absolute ethanol (5 mg/mL » 409 nmol) was added to the gallium-containing vial followed by the addition of acetate buffer with pH of 5.5 (450 μL). The mixture refluxed at 40° C for 25 min. The active solution was checked for radiochemical purity using ITLC and HPLC. The final solution was then passed through a 0.22μ m filter and the pH was adjusted to 5.5-7.

*Quality control of Ga-67 MAL*

*Radio thin layer chromatography*

A 5μ L sample of the final fraction was spotted on a chromatography Whatman No. 2 paper, and developed in mobile phase mixture, 10% NH_4_OAc and methanol 1 : 1.

*High performance liquid chromatography*

HPLC was performed with a flow rate of 1 mL/min and pressure of 130 KgF/cm^2^ for 20 min. HPLC was performed on the final preparation using a mixture of water : acetonitrile 3:2 (v/v) as the eluent by means of reversed phase column Whatman PartiSphere C_18_ 4.6 × 250 mm.

*Determination of Partition coefficient*

Partition coefficient (log *p*) of Ga-67 MAL was calculated followed by the determination of p (p = the ratio of specific activities of the organic and aqueous phases). A mixture of 1 mL of 1-octanol and 1 mL of isotonic acetate-buffered saline (pH = 7) containing approximately 3.7 MBq of the radiolabeled gallium complex at 37°C was vortexed 1 min and left 5 min. Following centrifugation at > 1200 g for 5 min, the octanol and aqueous phases were sampled and counted in an automatic well-type counter. A 500L sample of the octanol phase from this experiment was shaken again 2-3 times with fresh buffer samples. The reported log *p *values are the average of the second and third extractions from 3-4 independent measurements.

*Stability tests*

The stability of the complex was checked according to the conventional ITLC method (7). A sample of Ga-67 MAL (37 MBq) was kept at room temperature for 2 days while being checked by ITLC at time intervals in order to check the stability in final product using the above chromatography system. For serum stability studies, 500 μ L of freshly collected human serum was added to 36.1 MBq of Ga-67 MAL and the resulting mixture was incubated at 37°C for 5 h. The aliquots (5 μ L) were analyzed through ITLC.

*Biodistribution in Swiss mice*

The distribution of the radiolabelled complex among tissues was determined for Swiss mice immediately after the imaging. The total amount of radioactivity injected into each mouse was measured by counting the 1 mL syringe before and after the injection in a dose calibrator with fixed geometry. The animals were sacrificed using the animal care protocols at selected times after the injection (2, 4 and 24 h), the tissues (blood, heart, lung, brain, intestine, feces, skin, stomach, kidneys, liver, muscle and bone) were weighed and rinsed with normal saline and their specific activities were determined with a HPGe detector equipped with a sample holder device as the percent of injected dose per gram of tissues.

*Imaging of Ga-67 MAL in Swiss mice*

Images were taken 2, 4 and 24 h after the administration of the radiopharmaceutical using a dual-head SPECT system. The mouse-to-high energy septa distance was 12 cm. Images were taken from wild-type rats. The useful field of view (UFOV) was 540 mm × 400 mm.

## Results and Discussion

*Production*

Gallium-67, in form of GaCl_3_, was prepared through 24 MeV proton bombardment of the ^68^Zn target at Cyclone-30 on a regular basis. The target was bombarded with a current intensity of 170 μ A and a charge of 1400 μ Ah. The chemical separation process was based on a no-carrier-added method.

Radiochemical separation was performed through a two-step ion exchange chromatography method with a yield of higher than 95%. The quality control of the product was performed in two steps. Radionuclidic control showed the presence of 93(40%), 184(24%), 296(22%) and 378(7%) keV gamma energies, all originating from Ga-67 and showed a radionuclidic purity higher than 99%. The concentrations of zinc (from target material) and copper (from target support) were determined using polarography and shown to be below the internationally accepted levels, *i.e*. 0.1 ppm for Zn and Cu.

*Radiolabeling*

Due to the engagement of three MAL groups around Ga-67 core in complex structure, Tris (maltolato) Ga-67 (III) complex has a more lipophilic character. Chromatographic system was used for the detection of the radiolabelled compound from the free gallium cation. Using 10% NH_4_OAc and methanol 1:1 mixture, free gallium remains at the origin of the paper as a single peak, while the radiolabelled compound migrates to higher R_f_ (0.56).

Although the ITLC studies approved the production of radiolabelled compound, HPLC studies demonstrated the existence of radiolabelled species using both UV and scintillation detectors.

**Figure 2:**
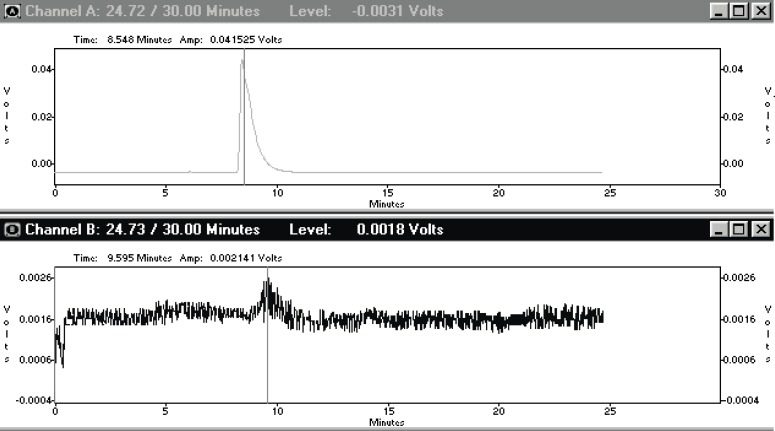
HPLC chromatograms of Ga-67 MAL on a reversed phase column using acetonitrile : water (40 : 60) (Up); (Down); scintillation chromatogram (Up); UV chromatogram

The eluting compound at 9.595 min (scintillation detector) related to 8.54 min peak (UV-detector) demonstrated a more lipophilic property of the complex. Free Ga cation was eluted at 1.02 min (not shown) (Figure 2).

*Partition coefficient of Ga-67 MAL*

As expected, the lipophilicity of the Ga-67 MAL compound is rather high. The measured octanol/water partition coefficient (p) for the complex was found to be depending on the pH of the solution. At the pH of 7 the log *p *was 0.40.

*Stability*

**Figure 3:**
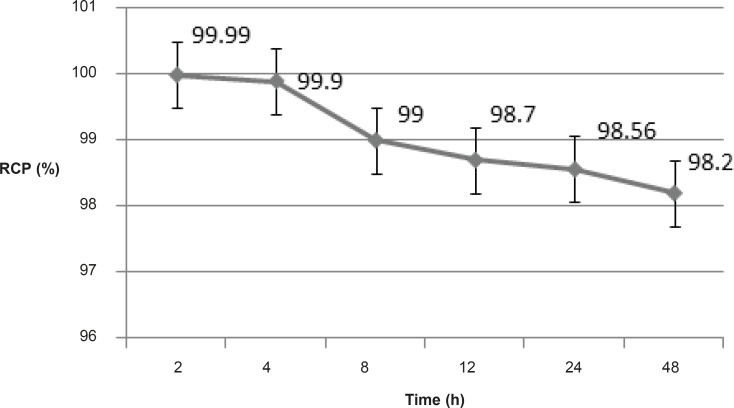
The radiochemical purities of a Ga-67 MAL sample 2-48 h at 37ºC in presence of human serum

The chemical stability of Ga-67 MAL was high enough to perform further studies. The incubation of Ga-67 MAL in freshly prepared human serum for 2 days at 37°C showed no loss of Ga-67 from the complex. The radiochemical purity of complex remained at 98% for 2 days under the physiologic conditions (Figure 3).

*Biodistribution in Swiss mice*

**Figure 4:**
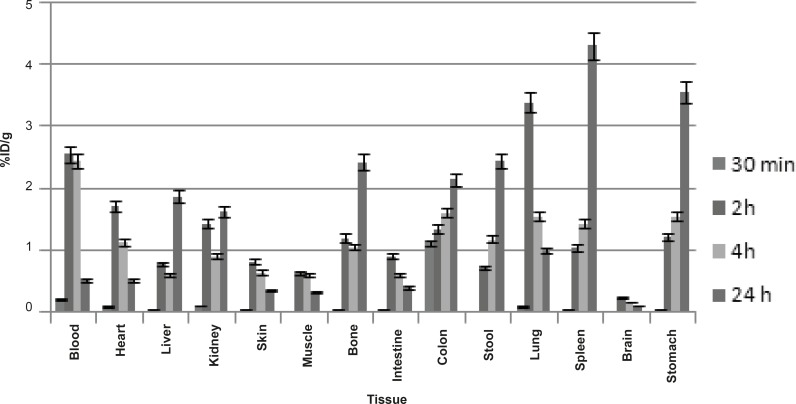
Biodistribution of Ga-67 chloride (1.85 MBq) in wild-type rats for 0.5-24 h after the IV-injection via the tail vein (ID/g %: percentage of injected dose per gram of tissue calculated based on the area under the curve of 184 keV peak in gamma spectrum) (n = 5).

For a better comparison, biodistribution study was performed for free Ga^3+^. The %ID/g data are summarized in Figure 4. As reported previously, Ga-67 is excreted majorly from gastrointestinal tract (GIT), thus, colon and stool activity content are significant while blood stream activity is high at 2-4 h followed by reduction in 24. Bone uptake is also observed after 24 h of post-injection.

**Figure 5:**
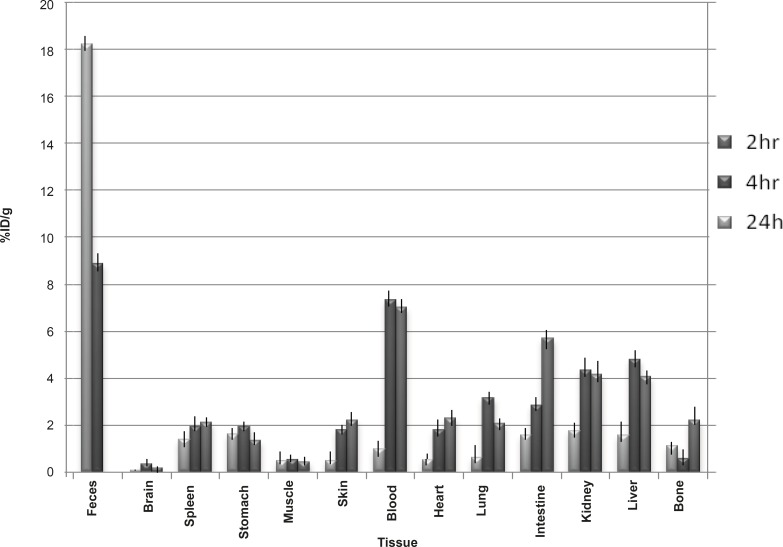
Biodistribution of Ga-67 MAL (1.85 MBq) in Swiss mice 2, 4 and 24 h after the IV-injection via tail vein (ID/g%: percentage of injected dose per gram of tissue calculated based on the area under the curve of 184 keV peak in gamma spectrum) (n = 3).

The radiolabelled compound biodistribution is also demonstrated in Figure 5. Due to the relative lipophilicity of the complex, the major activity in 2 h post-injection is present in liver, followed by rapid excretion through GI system (high stool content); the another major route of excretion for the labeled compound is urinary tract.

The comparison of vital organs uptake for Ga-67 MAL and Ga-67 chloride demonstrates the kinetic pattern difference for both species. Ga-67 cation is accumulated in the liver in the first 24 h post-injection slightly reaching a maximum after 24 h, while Ga-67 MAL is majorly accumulated in liver but then excreted.

As shown earlier, Ga-67 cation is slightly absorbed in the skeletal system (2-2.5%). On the other hand, the labeled compound almost shows fewer uptakes in the bone in 24 h after the injection.

Due to the high solubility of the complex, both in water and lipids, kidney is another excretory organ and shows high activity for the labeled compound especially after 4 h.

Both compounds are present in the blood up to 4 h, while in case of complex, it is more circulating due to the less excretion and liver extraction. Ga cation is rapidly captured by circulating metalloproteins (transferrin) and transferred to the liver.

Since the gallium cation can be trapped in blood cells due to the high content of Thio-proteins, the spleen activity content is increasing in this organ after 24 h compared to the radiolabelled compound.

*Imaging of Swiss mice*

**Figure 6:**
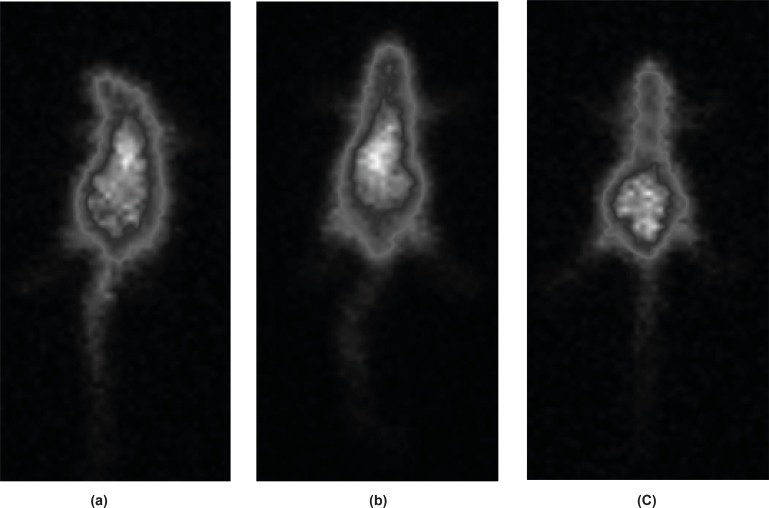
SPECT images of Ga-67 MAL (90 MBq, 22 μCi) in wild-type rats 2 h (a), 4 h (b) and 24 h (c) post-injection

Imaging in the mice showed a distinct accumulation of the radiotracer in the abdomen region all the time after the injection. Most of the activity is washed out from the body after 24 h by means of feces (Figure 6).

## Conclusion

Total labeling and formulation of Ga-67 MAL took about 30 min (RCP > 97% ITLC, > 98% HPLC, specific activity: 13-14 GBq/mmol). The complex was stable in final formulation and human serum at least for 24 h. At the pH of 7, the log *p *was 0.4. The biodistribution of the labeled compound in vital organs of Swiss mice was studied using scarification studies and SPECT imaging up to 24 h.

A detailed comparative pharmacokinetic study performed for Ga-67 cation and Ga-67 MAL. Gallium MAL is stable in aqueous solutions at pH of 5 to 8, and it has significant solubility in both water and lipids and hence the complex is mostly washed out from the circulation through the liver and kidneys faster than Ga-67 cation (or citrate) and can be an interesting agent for tumor detection for liver cancer and gastrointestinal cancers.

The biological evaluation of the tracer should be evaluated in tumor-bearing animal models. It is suggested that Ga-67 MAL could be a possible SPECT tracer, however, considering the complex fast wash-out and gallium-68 short half life, Ga-67 MAL can be a suitable candidate for tumor imaging applications and future Ga-68 PET studies and less use and therefore less imposed radiation doses to patients.
